# Weighing in: qualitative explorations of weight restoration as recovery in anorexia nervosa

**DOI:** 10.1186/s40337-023-00736-9

**Published:** 2023-01-31

**Authors:** Emily B. Barko, Sara M. Moorman

**Affiliations:** Department of Sociology, Boston College, 140 Commonwealth Avenue, McGuinn Hall 426, Chestnut Hill, MA 02467 USA

**Keywords:** Eating disorder, Diet culture, Health, Illness, Sick enough, Treatment, Weight stigma

## Abstract

**Background:**

Anorexia Nervosa (AN) continues to capture the public’s imagination, centered around physical appearance, particularly weight. Clinical conceptions of AN also emphasize weight. The objective of this study was to explore how individuals with lived AN experience thought about the role of weight in illness and recovery.

**Methods:**

The current study employed a grounded theory approach through qualitative inductive inquiry and analysis of 150 anonymous narratives, exploring firsthand experience of AN and recovery of adult individuals, based in the United States of America.

**Results:**

Individuals with AN histories contested intersecting popular cultural and medical presumptions of their health and illness positioned in weight. Respondents indicated that while weight does not *measure* recovery, it *matters* to recovery in unanticipated ways. Others’ expectations for a low weight served as a gatekeeper to various forms of social and institutional support. Respondents felt that the weight obsessions of other people made it difficult to earn the illness legitimacy to access sufficient care.

**Conclusions:**

Research findings bear implications for future AN research, advocacy, and clinical practice, as respondents pivot research emphasis from weight as a sociocultural motivation for AN, to weight as a sociocultural obstacle to AN recovery.

## Plain English summary

The manuscript describes the results of inductive, grounded theory analysis of 150 narratives, with the aim to explore what researchers most need to know about the role of weight in anorexia nervosa (AN) and recovery. Sociological theories of AN have focused on the sociocultural context in which people experience AN. We reframe that sociocultural focus using the perspectives of individuals with AN experience, who challenge weight and appearance-based understandings of AN. This study offers important insights surrounding perceived appearances of AN and recovery, motivations for AN, and barriers to healing. Voices of individuals with AN are important to hear to better understand recovery, low rates of treatment completion, and high rates of suicide in AN.

## Introduction

The primary professional diagnostic criteria for Anorexia Nervosa (AN) in the Diagnostic and Statistical Manual of Mental Health Disorders, fifth edition (DSM-5) are: (1) restricted eating, leading to a “weight that is less than minimally normal”; (2) “intense fear of gaining weight or becoming fat,” despite low weight; and (3) body image disturbance or denial of illness severity [2]. Individuals with AN often refuse treatment, which clinicians believe reflects the egosyntonic nature of the illness, alongside “delusional beliefs about body image, coupled with impaired judgement and cognition caused by starvation” [3, p419]. AN has the highest mortality rate of any mental illness [4–6]. Overall, AN is conceptualized as a drive to be thin at the potential cost of death.

What more is there to learn about the role of weight in AN beyond a portrait of “dying to be thin?” The present research centers individuals with AN history as the experts of their experience, from whom there is much for researchers to learn. Subsequently, this paper asks: How do individuals with firsthand AN knowledge understand weight loss and weight restoration in relation to recovery, and what do they believe researchers still need to know to better understand their experience? The importance of this study stems from an epistemological view that individuals who experience and narrate AN can be primary “knowers” or experts in this arena [7]. This research is urgent because conventional treatment practices, which may diminish personal voice in AN healing processes, may cause iatrogenic harm [8, 9].

### Prevalence and prognosis

Though modest in lifetime prevalence, affecting up to 4% among females and 0.3% among males, the illness’s impact is potent. Individuals may experience a mortality rate at least five times greater than the general population [10], with some estimates as high as a 16-fold [11]. When AN becomes chronic, deemed “severe and enduring anorexia nervosa” (SE-AN), individuals experience a significant decline in life expectancy, with a mortality rate of 20% at 20 years of illness [5]. Touyz and Hay [5, p2] indicate: “it is unfortunately not uncommon for death to occur in young adults in their thirties with a further 5–10% every decade thereafter” (2). Thus, early recognition and intervention are essential, with an average age of onset of 18 [12, 13]. Effects from starvation are a major cause of death, largely related to cardiovascular complications [14]. However, the second leading cause of death in AN, accounting for approximately 20% of deaths, is suicide [15, 16]. With so much attention to weight and the physical body in popular cultural depictions of AN, these suicide statistics are a powerful reminder of the psychiatric components of AN.

Treatment substantially mitigates the risk of death; however, there is a lack of an evidence base to set standards of care, and many people face difficulty in accessing treatment [17]. Almost half of individuals with eating disorders are insured through Medicaid in the United States of America (USA), which doctors rarely accept because of its notoriously low reimbursement rates [18]. Yet, Medicaid is often the only public insurance option for millions of low-income Americans [19]. Severe cases of AN are among the costliest mental illnesses to treat, and so private insurance companies also minimize coverage [20]. For instance, individuals with an AN diagnosis who may need more intensive care, such as hospitalization, can be denied treatment for weighing too much [21].

Definitional issues in AN experience extend beyond the weight requirements for accessing intensive care. Indeed, what constitutes “recovery,” beyond survival, remains undefined [22]. Remission criteria were added to the DSM-5 for the first time in 2013, and the manual does not employ the term “recovery.” Weight restoration is often the central DSM-5 criterion for remission, and sometimes the only criterion employed in empirical research [23]. Khalsa and colleagues [23, p2] maintain, “A greater consensus regarding the definition [of relapse, recovery, or remission] would be of considerable benefit to clinicians, researchers, patients, and family members, by allowing all constituents to speak the same language.”

If all constituents are to speak the same language, it becomes critical to ask, whose language will they speak? To this end, we center individuals with AN history to help better conceptualize AN and recovery through more nuanced understanding of the role of weight in AN. In the subsequent sections, we review relevant theory and research that postulates why weight matters in AN.

### Clinical criteria in sociocultural context

Theories about the etiology of AN play significant roles in how people—the general public and clinicians alike—comprehend and evaluate AN and recovery. Prior medical and mass media perceptions of AN have focused on it as an illness of weight, as a metric and/or aesthetic. Early debates (primarily of the late 1970s to early 2000s) broadly centered around whether AN was the result of internal psychological distress or the outcome of a misogynistic sociocultural context. The former largely focused on AN as weight loss as a strategy for psychological solace [24], while the latter allotted attention to the social rewards attached to thinness [25].

Contemporary research into AN is increasingly more interdisciplinary and dynamic in etiological inquiry and epistemological approach, for instance, in considering relationships between the social environment, individual psychology, neuroscience, and epigenetics [26, 27]. But even as academic theories have advanced to more complex explanations, AN is still conceptualized in reductionist ways in the cultural imagination, where appearance ideals continue to dominate [8, 28]. Legislative initiatives, including body mass index (BMI) minimum mandates for models and advertiser notes on altered images, imply that altering popular media will serve as a protective factor against AN [29].

No less than the National Institute of Mental Health [30] states, “Eating disorders are *actually* serious illnesses…” [emphasis added]. While this might seem like a trivial critique, for comparative context, it is unlikely that the National Cancer Institute would need to elaborate that cancer is actually serious. Warin [31, p9] noted how “people dying of cancer are rarely represented in the same exhibitionist manner” as people dying of AN. Media has aimed to lure sociocultural fascination through “shocking color images of young women’s emaciated bodies” (9). These extreme manifestations of AN, of “carnivalesque” imagery and “enticement of spectacle” [31, p9] can distort AN experience in ways we emphasize in this paper.

Further complicating matters is the extent to which thinness is not only idealized as a fashionable aesthetic, but also as a personal health achievement [28]. Two out of three Americans have a BMI in the “overweight” or “obese” range, and the U.S. has 72 billion-dollar diet and fitness industries, earning it the label “a cult of thinness” [32–35]. The prevalence of negative perceptions of higher-body weight clients among physicians is well-documented, with “higher-body weight clients being deemed ‘lazy,’ ‘sloppy,’ having ‘poor self-control,’ ‘unattractive,’ ‘indulgent,’ and ‘noncompliant’ [36]. In contrast to a stigmatized fat body, a thin body is often positively perceived, even if that body is sick [37, 38]. In AN, low weight becomes distinct from other symptoms such as anxiety and depression, given the often-lauded physical and psychological traits attached to thinness, such as discipline [39]. Sociologist Sharlene Hesse-Biber [34, p94] has asserted “disorderly eating is not a sign of pathology, but a strategy that is a ‘normal’ part of the female existence,” and broader human experience [34, 35]. What makes people with AN labelled as pathologically ill is that dieting, fear of weight gain, and body image disturbance occur in a body that is at “a significantly low weight” [2]. Whether and when weight fixation is pathological evolves as a theme throughout this paper.

Notably, these oversimplified and overgeneralized understandings of AN as beauty or celebutante aspiration can feel invalidating to people with AN, even interfering with getting well [40]. Even doctors, both within and beyond eating disorder specialty healthcare settings, may be heavily swayed by misleading conventional conceptions of AN [36]. For example, when mental health trainees saw clients with higher body weights, they minimized those clients’ symptoms, referred them for fewer therapy sessions, and attached negative stereotypes to their descriptions of the clients [41]. Trainees may have little other context: In “A National Survey of Eating Disorder Training” the authors found that of 637 responding medical programs, only 19% scheduled or had elective rotations for eating disorders [42]. Moreover, internal medicine averaged just 1.94 h of teaching about eating disorders, and even general and child/adolescent psychiatry averaged only 4 h [42]. Given that clinicians, as human beings, are not free from bias, stereotypes of eating disorders can influence illness assessments. Healthcare providers’ understandings of AN as trivial and choice-driven can make them reluctant to offer treatment [40]. For example, health care professionals often fail to offer higher-weight individuals the same level of care that they offer to individuals in lower-weight bodies even when the physical effects of malnutrition are similarly pronounced [43].

In sum, both mass media and medical perceptions have focused on AN as an illness of weight, as a metric and/or aesthetic. Broadly, less weight is believed to constitute more illness, while more weight is deemed less illness [43]. Given the high rates of suicide attempts and completed suicides in AN, this continued concentration on weight implies possible misunderstanding of AN experience [16]. To this end, in this study, we explore how weight loss and restoration are conceptualized from subjective perspectives of AN experience, and, in turn, ask what respondents believe researchers most need to know for better understanding.

## Data and methods

### Participants

Given situational power dynamics surrounding what constitutes knowledge, who has a diagnosis, which experiences are legitimate, and by what metrics, the majority of AN narratives have not yet been heard [44, 45]. Consequently, this research holds that preestablished professional expertise should not be the only knowledge source to critically inform how to understand AN. We access subjugated knowledge in centering lived experience as a subversive form of expertise. This methodological pivot may hold heightened relevance for an illness where individuals are “not always trusted to be ‘accurate’ tellers of their stories” [45, p707].

This study was approved by the Boston College Institutional Review Board at the researchers’ home institution. In 2017, using Facebook and Instagram, we recruited volunteers aged 18 and older who self-identified as “someone who has had experience with AN.” The first author posted a call for participants and link to the study to their accounts and asked others to share this information; therefore, recruitment was by snowball sampling.

We received 150 responses, from 148 participants who identified as a woman, 1 who identified as a man, and 1 who selected “other/not applicable.” (We subsequently use gender neutral pronouns to be more inclusive when discussing the sample.) Most participants (137) identified as “White,” seven as “Asian,” five as “Other race/ethnicity,” and one as “Black.” Participants were diverse in income (see Table 1).

**Table 1:** Sociodemographic Characteristics of Study Participants and Source of AN Label

	% of sample	*N*
*Race/Ethnicity*		
White	91	137
Asian, Black, or other race / ethnicity	9	13
*Annual Income ($)*		
Less than 30,000	15	23
30,000–59,999	14	23
60,000–99,999	18	26
100,000 or more	34	51
No response	18	27
*Highest Level of Education*		
Less than high school or high school /graduate equivalency degree	10	14
Some college	37	56
Associate’s or bachelor’s degree	31	47
Master’s, doctoral, or other professional degree	22	33
*Age Group (years)*		
Under 20	17	25
20–24	38	57
25–34	27	40
35–44	11	17
45 or older	7	11
*Source of AN Label*		
Psychiatrist/psychologist	35	53
Primary care physician	23	34
No response	23	34
Family, friend, or other person	9	15
Self	9	14

We were interested in demographic data on socioeconomic status (SES), including household income and level of education given SES is “a consistent and reliable predictor” of a multitude of both physical and psychological health outcomes [46]. Likewise, SES predicts the likelihood an individual would be diagnosed with AN and/or have access to treatment [47]. While eating disorders impact individuals across SES groups, given “affluence stereotypes” of eating disorders, individuals with lower SES are less likely to have access to screening, adequate level of care in clinical assessment, and subsequent treatment opportunities [47, 48]. Notably, racism also impacts disparities in healthcare [47–49]. Thus, intersectional analyses of participant demographics help to better contextualize the voices of our participants, and the privileges that may afford research presence for some individuals over others.

Most participants were highly educated, and the majority of participants were older than adolescence.

Allowing participants to conceptualize what “experience with AN” entailed was a principal part of the research. We asked participants, “Your diagnosis of anorexia nervosa was determined by…?” with the closed-ended options of self; primary care physician; psychiatrist/psychologist; family; friend; or other. A medical professional was the most common response (see Table 1).

### Narratives

Respondents completed an online form comprised primarily of open-ended questions. We believe that the anonymity of the mode and the lack of any face-to-face interaction were strengths in creating a disarming and nonjudgmental setting for respondents to share more personal information than they might face-to-face. Given that highly sensitive topics may elicit feelings of “shame” and/or “fear or exposure,” “anonymity rather than mere confidentiality,” may be requisite for participation [50, p21]. Anonymity can also encourage individuals to be “unusually forthcoming and helpful” [51, p5]. Consequently, we believe this approach strengthened the credibility and trustworthiness of the data given that “sharing personal information and divulging secrets more frequently than in face-to-face communications is one of the most consistent findings of anonymity studies” [51, p5]. Further, we considered that respondents may have had previous disempowering evaluations of their bodies and appearance [21, 52]. Respondents may bear heightened sensitivities towards power dynamics in methods that include in-person interactions and settings that emphasize researcher authority.

Individuals with AN do not always consider clinical AN and recovery metrics to be valid or reliable, so we did not ask about them [52, 53]. We did not ask for reports of current or former weight. Instead, we presented six narrative prompts where participants could write as much or as little as they wanted. In this paper, we primarily analyzed responses to three of those prompts, which inform the content for each narrative:

1. Drawing on your experience, what do you think is most important for eating disorder researchers to know or to better understand about experience with *anorexia nervosa*?
2. Again, drawing on your experience, what do you think is most important for eating disorder researchers to know or to better understand about experience with *recovery*?
3. Some people argue that weight restoration is the best way to measure recovery—while other people strongly disagree that weight corresponds to health. Please comment on your stance, if any, on this debate.

Some participants wrote relevant answers to these questions in response to the other three narrative prompts, and we analyzed that text as well. Notably, in our last survey question, we invited respondents to reflect upon aspects of AN and recovery experience we may have overlooked. We asked respondents to “share any additional thoughts [they] feel might be important for eating disorder researchers to consider for improved understandings of anorexia nervosa and/or recovery.” While we could not ask participants to confirm the credibility of our analyses given their anonymity, we believe the open-ended inquiry, and thematic congruence in responses across questions likewise reinforced strong content validity and data trustworthiness.

### Analytic strategy

The first author conducted the qualitative analysis, with an inductive approach and using grounded theory as a method, approach, and a theoretical process [54]. Overall, grounded theory served as a constant comparative method employed to reconfigure the data pieces to discover a more complete whole [55–57]. With the goal to derive a general abstract theory of process, or action, or interaction, grounded in the views and direct narrative responses from the participants in the study, we analyzed the data, inducing theories directly from the narrative responses [54, 58]. We began the dynamic and iterative process of coding and memoing in order to both describe and analyze the data and to continue to develop and reconfigure patterns of descriptive codes and evolutions of analytical codes [56, p335]. While centering an inductive iterative approach to analysis, interpretation, and theoretical generation, these processes also included initial inductive coding, recoding, memoing, and perpetual provisional theory re/generation [56]. These central analytic aids allowed us to develop concepts, themes, and categories in the data from our broader inquiries [54, 56]. For example, processes of analysis included reading over the material and applying line-by-line open codes to describe what the participant was relaying in their response. This approach included analyzing inquiries first within each open-ended survey question, and subsequently across questions.

Beginning with descriptive codes, we later moved towards more analytical concepts and categories, which became our theoretical codes. While we conducted initial coding manually, we also used automated coding with Microsoft Word to ensure we did not omit concepts and themes. Reflexively, we scrutinized biases we might hold to ensure we did not unintentionally exclude themes we might initially consider to be less compelling. For example, after several rounds of inductive analyses, we conducted word counts for key terms that we did not find through inductive literal and thematic coding that prior research would anticipate, e.g. AN motivated by appearance ideals. In the same way, we took extra care to look for negative cases for central phenomena that we did identify to ensure that we were not merely assuming an absence of exceptions or nuances.

Our goal throughout this research process has centered on an aim to gain insight and understanding into lived experiences of AN recovery. Indeed, from a “constructive interpretive approach,” we have attempted to comprehend the subjective experiences that comprise the realities for participants [56, p77]. We believe that it can be a therapeutic experience to be able to verbalize one’s own thoughts, perceptions, and concerns in relation to self and society. Such an opportunity may especially resonate with respondents who have felt isolated in AN, and/or not heard by treatment providers [9]. Likewise, it may also be an empowering experience for a participant to be viewed and accepted as an expert in one’s own right to the researcher, specifically in an arena of AN experience where health providers often deem impaired insight to be a common feature in self-evaluation [59].

## Results

Respondents spoke to two predominant themes, including: (1) why weight does or does not *measure* AN and recovery, and (2) how weight does and does not *matter* in AN and recovery. Participants called attention to professional evaluative factors that shaped and interacted with their personal experience. Collectively, respondents offered compelling insights into weight obsessions in AN and recovery, catalyzing critical reconsideration of what is, and is not, rational.

### Weight as a measure

DSM-5 criteria operationalize AN as abnormally low weight accompanied by fear of weight restoration and denial of the seriousness of the problem. Participants countered each of these criteria. They did not try to defend low weight as healthy, or contend that a healthy weight was an unnecessary component of recovery. Rather, one described weight restoration as “necessary for healing and nourishing the brain.” Respondents positioned the importance of weight restoration in healing in the following quotes:

“There is no way to [be] recovered without being weight restored. If you think you’re recovered but you’re still at a low body weight, you’re fooling yourself.”

“Although the eating disorder voice in my head wants me to say that weight restoration doesn’t matter, I know it does. Your body and mind are connected. If your body isn’t in a healthy place, then your mind cannot be in its healthiest place…”

“I believe that weight restoration is necessary to the point where the body and brain are no longer malnourished and function in a healthy way.”

Readers may perceive that these quotes preceded caveats, and they did. Participants argued that AN is an extremely serious illness, but not one that is solely or even primarily about low weight. Respondents begged researchers to take AN seriously, regardless of weight. One respondent emphasized that “It’s a MENTAL illness.” Respondents reiterated again and again the importance of the mental components of AN in recovery, which they believe are underappreciated through hyper-focus on the body. Another participant implored, “Teach people in textbooks and literature about the truth when it comes to eating disorders. It’s not about the weight, it’s about control and so many other issues.” Numerous respondents wished to highlight that when focusing on weight, clinicians risk overlooking very sick people whose weight is not—or is not *yet*—dramatically low:

“YOU DON’T HAVE TO BE SKIN AND BONES TO BE REALLY SICK!”

“[I would like researchers to understand] the lack of correlation between psychological/mental health and physical health (specifically weight).”

“I would like researchers to understand that weight should not be seen as the most important measure of the degree of severity of anorexia nervosa. Many people that suffer do not always drop to extremely low weights, but that does not mean that their eating disorder is any less significant. Researchers need to remember that weight is only a physical representation of a mental illness.”

Therefore, respondents argued strongly that weight restoration is necessary but not at all sufficient for recovery. Many participants referenced occasions when they were very ill, despite weighing a medically-acceptable amount. They described lingering psychological distress:

“I have been in my worst mental state ever at my highest weight. You couldn’t tell from my weight that something was wrong.”

“There are underlying mental illnesses and traumas in many of us that need to be addressed before we can recover. [Weight restoration] would be like putting a band-aid on a broken arm; if the arm is not set, the band-aid does nothing. Please, focus on finding out WHY people developed Anorexia and fix it. That would help so many of us recover.”

“Weight restoration is not a proper or standard for measuring recovery. After the weight is gained, the eating disorder does not suddenly vanish, after all this is a mental illness. A person may be weight restored but still suffering from the deep-rooted issues that are not physically manifested.”

“Weight plays an important role in medical stability but as a measure of overall recovery it is grossly inadequate. Someone can be weight restored but still be committed to their ED and still struggle with the same stressors, or lack effective coping techniques.”

From respondents’ viewpoints, it is important for researchers to know that there is not always a positive relationship between weight gain and recovery. Individuals ardently exclaimed: less weight did not always indicate more illness, and equally, more weight did not always indicate more recovery. In other words, while weight gain might conceal AN, it does not eliminate the illness essence. Respondents insinuated the dangers of a “misaligned” AN presentation that does not meet the expectations of others:

"Now I just feel like an anorexic, but no longer look like one.”

In this statement, the participant was not expressing a continued personal desire to resume looking “like an anorexic.” Rather, in the second theme, respondents illustrated how appearance matters because the pervasive understandings of social others—including doctors, clinicians, researchers, peers, insurance companies—have staggering impacts on their illness experience. While respondents previously debunked the validity and reliability of weight as a measure of illness state, they subsequently revealed how when it comes to healing, weight—looking like an anorexic—still matters.

### Weight matters

Participants described weight restoration—what they meant when they used the term “medically stable”—not as the end of recovery, but rather as the point at which their recovery began to take off:

“Weight restoration is the first of many steps in recovery from anorexia. There's still all the mental stuff to take care of.”

“I was weight restored but at my worst after four months of inpatient treatment. The struggle to recover truly begins when you decide to recover, but the support seems to end at that most crucial point.”

“Researchers need better understanding of the recovery process and amount of treatment needed so that insurance isn’t as huge of an obstacle—especially putting more focus on the psychological aspects of recovery so that insurance/doctors keep providing necessary treatment to anorexia sufferers even after they are more medically stable.”

However, as two of the above participants referenced, other people saw them as recovered once they regained a normal weight. Accessing social support became contingent upon the ways that weight mattered to others.

“Once weight-restored, nobody wanted to hear about my ED.”

“Being in an eating disorder recovery hospital it's hard to stay once you have been weight restored because people automatically think you are healthy again.”

“Imagine everyone thinking that things you struggle with every moment of every day is a big joke and that gaining weight will solve it.”

Others’ perceptions determined not only access to informal social support, but also access to formal medical care. In particular, most insurance plans only authorized formal treatment for underweight patients, and withdrew that authorization once the individual was weight-restored. To continue treatment, individuals and their families would have had to pay out-of-pocket for care, an option that is prohibitively expensive for most.

“[Eating disorder researchers should know] that someone's BMI/weight does not necessarily mean they are healthy, i.e. insurance providers will stop paying for treatment once [a] patient hits a certain weight, even if that said patient is still struggling mentally and physically.”

“I know some treatment centers will send patients home as soon as they hit their recommended goal weight - and I think that's a disaster plan for relapse. (From my own experience).”

“All eating disorders are serious and weight should not be used as a measurement of a person’s suffering, nor should it ever be used as the main determining factor for level of treatment as many insurance companies currently do. I also think researchers should work with insurance companies so that they can better understand the impact of their level of care determinations have on the patient. I have personally met many people that felt that because an insurance company denied them treatment based on their weight, they were ‘not sick enough.’ These people then become sicker and sicker and end up in treatment with more damage and more complications both mental and physical than they would have if their insurance company had acted when they first pursued treatment.”

In order to qualify, or re-qualify, for treatment, some participants reverted to behaviors they knew were dangerous:

“I was weight-restored against my will for years on end and would leave a hospital or treatment center and go right back to my anorexic behaviors.”

“So I dropped the weight [again] and suddenly it became acceptable for me to get help. My body was my voice.”

These participants *did* fear weight gain, as the DSM-5 diagnostic criteria said that they would. But they did not fear it due to their concerns about appearance. Many participants were openly derisive of that idea:

“I often see articles and literature overemphasize the reasoning behind anorexia as a desire to look better or that a sufferer of the disease simply has a distorted view of themselves; I never looked in the mirror and saw myself as obese, like the recurring tropes in the media suggest.…”

“Nobody looks at a billboard of a hot girl and goes ‘Aw fuck, I guess I have to develop a life long illness now…’”

“Anorexia isn't just a look, nor is it a fad; anorexia has destroyed me and will continue to destroy me until I die.”

Rather, participants feared weight gain because they feared losing access to treatment.

Participants also commented on the ways in which institutional gatekeeping and others’ perceptions of weight had restricted their access to much-needed early intervention in the beginning of their illness.

“Patterns and symptoms need to be better recognized in those who many not be underweight, yet. I personally went from overweight to almost underweight in a year and it was not recognized by anyone, except a close friend who was in recovery for [anorexia].”

“The eating disorder becomes your life. It is very hard to get out of the mindset once you are in it.”

“I think there is a much greater opportunity for early intervention in the world of anorexia and other EDs [eating disorders]. My experience was such that attention paid to eating disorders was largely focused on only anorexia and only resulted in intervention at a point where the situation was particularly severe and obviously visible. I know for myself personally, having not been considered a ‘severe’ case, I did not nor did those around me push intervention as an option until I was much further down the road. I believe that made the recovery particularly difficult and drawn out in my case.”

In short, from respondents’ perspectives, far too much weight was given to weight, which made “fear of weight gain” as a diagnostic criterion seem positively rational.

## Discussion

We have centered subjective experiences of illness in order to explore what researchers most need to know about AN in relation to weight as a metric of recovery. Drawing upon narrative responses from 150 individuals, we found that respondents depicted weight as a foundationally deceptive starting point for defining AN given it is a *physical* measure of a *mental* illness. While respondents believed that weight did not measure AN well, they did acknowledge that it mattered in recovery, both as a first step towards healing and because low weight became requisite to access and sustain support. Sometimes weight loss paradoxically became the most effective way to access recovery.

Sociology has a rich tradition of theoretical and empirical work on stigma and visibility in illness [60]. Application of these ideas to AN has largely just begun, despite the undeniable relevance of appearance and body image. Our results complement those of prior work. In the work of Federici and Kaplan [52], participants (selected by BMI from among hospitalized patients) reported that a contributing factor to relapse was that their AN was no longer taken seriously following weight restorative treatment. With interviewees who had been diagnosed with AN, Eiring and colleagues [21] initiated critical dialogue exploring how stereotypes of AN feed into ‘not sick enough’ consequences, including dangers that materialize from “proof” of illness confirmation that becomes requisite for support. Their findings, in tandem with this current study, offer critical nuance to a perception that “the tragic and nearly universal reality for those with eating disorders is that they often believe they aren’t sick enough to warrant changing their behaviors or seeking help” [61, p4]. Rather than “not being sick enough” as a hallmark component of AN as a mental illness, respondents called attention to “not sick enough” as emblematic of situational dynamics that present skepticism to the gravity of AN, especially when one does not “look the part.”

Importantly, our study joins an unconventional body of work in which individuals with AN have reported on their experience without qualification. While many researchers ask about participants' first-person experience, the overwhelming majority of studies only enroll participants who qualify according to conventional metrics, such as their weight/BMI or a formal psychiatric diagnosis. This approach may exclude less visible representations of AN, limiting exploration to illustrations of AN deductively anticipated. The current research has extended space for inclusion of individuals who may never have access to treatment, or may otherwise be omitted from AN research for body weights deemed too high or low for respective AN or recovery inquiry. Echoing Warin [31, p9], we “discard thinness as the definitive bodily marker of anorexia,” as “thinness denotes a static and fixed occupation of space and time.” To the contrary, individuals with AN have depicted variable, temporal bodies that do not always shift in accord with others’ expectations of health or illness.

Together, these findings lead us to implications for research, policy, and practice. First, researchers might recommit focus to the mental, rather than physical, aspects of AN. Second, health policy scholars should investigate whether current diagnostic criteria and restrictions on access to care produce iatrogenic harm. Third, clinicians might realize more positive outcomes among their clients if they reconceptualize the patient’s role in recovery. We discuss each of these points below.

### AN Is a mental illness

Voices of AN experience draw needed attention to a critical obstacle to recovery, the less visible mental health components of the illness that do not receive the same attention in research as does weight. This is even as psychiatric components of AN may prove to be no less fatal for those who die as a result of this illness [62]. Indeed, a recent meta-analysis of 35 randomized controlled trials of AN treatments showed that they achieved an increase in weight, but no effect on psychological outcomes [63].

A belief in a better future, measured holistically, might be a particularly important treatment outcome given aforementioned suicide statistics in AN [16]. While AN “has clearly delineated biomarkers of disease progression, for example bradycardia and raised liver enzymes” [5], perhaps quality of life markers could comprise additional psychological measures of AN or recovery progression. Such an approach would coincide with the work of Mitchison and colleagues [64] who found utility in “quality of life” as a measure in eating disorder recovery. Further, relapse prevention strategies may be strengthened by seeing beyond ‘just eat’ strategies for recovery, alongside decreased general public fixation on weight, given these cited elements as obstacles to recovery [40, 52].

### Iatrogenic harm

Scholars have recently criticized AN treatment on multiple grounds: recovery processes are of long duration, half of patients relapse, and AN becomes a chronic condition in approximately one out of five patients [17]. Kaye and Bulik [17, p591] continue: “Binding standards of care for AN do not exist. … Outcome data are rarely published. Marketing strategies are unconstrained by legislation and policy changes that limit the pharmaceutical industry.” Nevertheless, clinicians view treatment as essential [65].

Continued deliberation needs to be given to the ways that current DSM-5 criteria, as well as public and private insurance criteria in the USA, centers low weight as a requisite for diagnosis and treatment. Certainly, weight stigma hindering access to eating disorder treatment for individuals in higher weight bodies is not limited to the USA [66]. Requiring a certain weight for treatment may cause iatrogenic harm if people set that weight as a goal, refuse to gain weight above it, or more simply never weigh so little that they qualify for treatment. A fear of fatness or weight gain does not necessarily indicate a desire to be thinner as a component of mental illness, but may, as with our respondents, indicate a fear of losing support [67]. Time-delays matter: There is evidence that outcomes are better when intervention occurs in the first three years of illness [68], deceasing time between onset, detection, and treatment [69, 70]. Greater awareness of diversity in eating disorder presentation (beyond stereotypes) may better close these gaps [71]. While atypical anorexia (AAN), or restrictive eating in individuals with normal BMI, may now be more common than “typical” AN, individuals with AAN are significantly less likely to get treatment than “typical” patients [72]. For individuals with AAN who do acquire treatment, it is often through a longer delay: 11.6 years for AAN versus 2.5 years for AN [38]. In 2017, Claire Mysko, Chief Executive Officer of the National Eating Disorders Association in the USA, reported that two-thirds of people with an eating disorder never receive treatment [73].

Among the minority of individuals who have been able to access treatment, many have not felt seen and heard. Ramjan and Fogarty [9] found that only 25% of participants believed their treatment providers effectively listened to and understood their concerns. Other respondents have perceived providers to underestimate their efforts to get well if they struggled to improve following a ‘one-size fits all’ treatment approach [74, p5]. Furthering gaps in AN communication, individuals with AN are not always deemed credible in research studies, such that researchers conclude patients must be in denial when their results are unexpected, (e.g. respondents do not present body image disturbance as anticipated) [8].

### Ownership over recovery

In mental health and illness more broadly, the “recovery movement” offers the person with illness more ownership over recovery [75]. The recovery movement has served in part as a response to paternalism in medicine, which may be a particular problem in AN, an illness where individuals are often deemed to have problems with theory-of-mind and judgment as a symptom of the illness itself. Arbel and colleagues [59] found that while individuals with AN often do have some degree of true unawareness about their condition, perhaps half of instances labelled “poor insight” in fact involve the individual’s conscious disagreement with clinical judgment. Coerced or forced treatment may cause its own iatrogenic harm [76, 77].

The quality of the therapeutic relationship may matter more than the treatment approach itself [78]. Johns and colleagues [79] have written about the “power system” within AN treatment, where patients perceived opposition between themselves and their care providers, with power lying with the care provider. Johns and colleagues [79] recommend treatment paradigms that more highly value patients’ treatment goals, such as psychological well-being. Other authors are beginning to write about “palliative psychiatry,” in which improving patient-perceived quality of life, rather than reversing physical problems, is the primary goal of care [80].

### Limitations

This study has several limitations to consider. First, the disproportionate number of white women aged 34 and under largely reflects current commonly reported demographics of AN [46]. However, stereotypes of AN may cause self-selection and exclude males, people of color, and people in midlife or older age [45]. Consequently, interrogating the validity of the statistics, both in this current study, and in the diagnostic population at large, is important, as to avoid further entrenching the myth that eating disorders are an illness of white women [71].

Second, we neglected to ask participants how long they had been experiencing AN. All participants were legal adults, and the content of their disclosures about prior treatment suggests that many had years of experience. Some attitudes, such as acceptance of the importance of weight restoration, may not have emerged in a sample that was earlier in the course of AN.

Third, recruiting through AN groups on social media enhanced response, but may have resulted in a select sample. Individuals who do not affiliate with these groups had no way to learn about the study. Many individuals with AN are socially isolated, which means that belonging to an AN group may be the exception rather than the norm [81].

## Conclusion

By way of questioning weight-centric paradigms of AN, in both research and practice, a final contribution of this research pivots research emphasis from weight as a sociocultural motivation for AN to weight as a sociocultural obstacle to recovery. We have learned weight *matters,* but does not necessarily *measure* (AN or recovery). Therefore, how weight matters, shifts our attention (and needs to continue to shift our attention) to how weight matters for others: individuals, interactions, and institutions around the person with AN. Accordingly, sociocultural perspectives on eating disorders have astutely called attention to disordered contexts in which AN occurs, which in effect, expands illness beyond the individual, as well as questions the true health of “healthy” populations [35, 36]. Yet, while there has been valuable sociocultural emphasis on body image and ideals of attractiveness in understanding AN [25–28], findings indicated that: (1) thinness as beauty currency was by and large overtly dismissed and noted as a stereotype that keeps AN trivialized and misunderstood, and (2) sociocultural expectations for an AN appearance served as a gatekeeper for help, even in clinical arenas. Implications are that while appearance is assumed to hold great importance to people with AN, this research finds the significance of appearance in AN *to others* in need of further attention for more efficacious clinical treatment, academic research, and everyday understanding. In effect, for the respondents in this study, sociocultural expectations for AN appearance may have hindered recovery more than they contributed to illness development. While AN experience may continue to be depicted as “dying to be thin,” greater recognition of individuals who are dying to be thin (enough) in order to get well, offers another portrait worth further consideration.

## Data Availability

Due to the confidential nature of our data, and agreement with the Boston College Institutional Review Board, we are not able to publicly share our data.

## Abbreviations

AAN: Atypical anorexia nervosa
AN: Anorexia nervosa
DSM-5: Diagnostic and statistical manual fifth edition
BMI: Body mass index
SE-AN: Severe and enduring anorexia nervosa
